# Editorial

**Published:** 1874-05

**Authors:** 


### Editorial.

"Oh wad some power the giftie gie us, to see oursels as ithers see us." So sang auld Scotia's bard, and we have often mentally re-echoed the prayer, which has most unexpectedly been answered by our "Country Correspondent" in his "Review of the CHICAGO MEDICAL JOURNAL, April No." Long familiarity with the style of our "Correspondent" has convinced us of his entire exemption from the vice of flattery, and therefore we accept his commendations and condemnations with all due humility, hoping at some future day to merit the one and to escape the other entirely. Being human, we do not claim perfection, only aspiring thereto. May its partial attainment, with a little assistance from our "Correspondent," be not unattainable! H.

